# Particularités de la cardiomyopathie du péripartum en Afrique: le cas du Togo sur une étude prospective de 41 cas au Centre Hospitalier et Universitaire Sylvanus Olympio de Lomé

**DOI:** 10.11604/pamj.2014.17.245.3058

**Published:** 2014-04-01

**Authors:** Machihude Pio, Yaovi Afassinou, Soodougoua Baragou, Edem Goeh Akue, Souleymane Péssinaba, Borgatia Atta, Koffi Ehlan, Amouzou Alate, Findibe Damorou

**Affiliations:** 1 Service de Cardiologie, Centre Hospitalier Universitaire Sylvanus Olympio (CHU SO) de Lomé; 2 Service de Cardiologie. Centre Hospitalier Universitaire Campus de Lomé, Togo

**Keywords:** Insuffisance cardiaque, cardiomyopathie, péri-partum, facteurs de risque, heart failure, cardiomyopathy, peri-partum, risk factors

## Abstract

**Introduction:**

La cardiomyopathie du péripartum (CMPP) est une défaillance cardiaque dont l’étiologie demeure encore méconnue.

**Méthodes:**

Il s'agit d'une étude prospective descriptive réalisée dans le service de cardiologie du CHU Sylvanus olympio de Lomé du 1er janvier 2010 au 30 avril 2012. Elle a concerné 41 patientes ayant présenté une insuffisance cardiaque entre le 8eme mois de la grossesse et les 5 premiers mois du post-partum.

**Résultats:**

L’âge moyen des patientes était de 31,47 ans (extrêmes 21 et 44ans). L'incidence de la CMPP était de 1/362 grossesses. La parité moyenne était de 3,07 (extrêmes 1 et 6). Les symptômes étaient apparus dans le post-partum dans 90,24% des cas. Un retard important de diagnostic était observé. L'insuffisance cardiaque globale était le mode de décompensation dans 65,85%. Les signes électrocardiographiques étaient essentiellement la tachycardie sinusale (97,56%) et l'hypertrophie ventriculaire gauche (97,56%). L’échographie cardiaque a montré dans tous les cas une cardiomyopathie dilatée. Quatre cas de thrombus intraventriculaire gauche étaient notés. La FEVG était sévèrement altérée. L'HTAP était importante dans 56,09%.

**Conclusion:**

La cardiomyopathie du péripartum est une complication cardiaque grave de la grossesse de cause inconnue, fréquente dans la population africaine.

## Introduction

La cardiomyopathie du péripartum (CMPP) ou syndrome de MEADOWS est une défaillance cardiaque apparemment primitive à cœur dilaté survenant entre le huitième mois de la grossesse et les cinq premiers mois suivant l'accouchement chez des femmes à cœur antérieurement sain [1, 2]. C'est une pathologie dont l’étiologie reste encore méconnue de nos jours d'où son nom de cardiomyopathie idiopathique ou primitive [3, 4]. Son incidence est très variable mais l'affection serait plus fréquente chez les femmes de race noire et particulièrement en Afrique noire [5, 6]. Pourtant les données de la littérature font totalement défaut au Togo sur la CMPP. Il nous a donc paru nécessaire d’étudier la CMPP dans ses aspects épidémiologiques, cliniques et paracliniques.

## Méthodes

Il s'agit d'une étude transversale descriptive qui s’était déroulée sur une période de 27 mois (1^er^ janvier 2010 au 30 avril 2012) dans le service de cardiologie du CHU Sylvanus olympio de Lomé.

Ont été inclues dans l’étude des femmes quelque soit leur âge, leur race qui ont présenté une insuffisance cardiaque(IC) entre le huitième mois de grossesse et les cinq premiers mois du post-partum sans étiologie retrouvée et chez qui une cardiomyopathie dilatée (CMD) a été diagnostiquée à l’échodoppler cardiaque. N'ont pas été inclues dans l’étude les femmes présentant un début d′IC avant le huitième mois de grossesse ou après les six premiers mois du post-partum, les femmes ayant une cardiopathie connue ou toute autre cause d'IC.

Une fiche d'enquête préétablie a servi de recueil des données démographiques, les données des carnets des consultations prénatales, des facteurs de risques cardiovasculaires, le mode de survenue ou de décompensation de l'IC, le mode et le déroulement de l'accouchement. La chronologie des signes de l'IC par rapport à l'accouchement, et les données de l'examen physique et paracliniques. Nous avons demandé systématiquement la radiographie du thorax, l’électrocardiogramme, l’échodoppler cardiaque, la numération formule sanguine, la glycémie, la créatinémie, l'uricémie, la protéinurie, l'ionogramme sanguin, la sérologie HIV. Les d-dimères, les enzymes cardiaques et l'angioscanner thoracique étaient demandés en fonction du contexte clinique et électrique

Outre l'absence de cause de l'insuffisance cardiaque, les critères échographiques suivants étaient indispensables pour retenir le diagnostic de CMPP: la dilatation au moins du ventricule gauche (DTDVG> 32mm/m^2^ de surface corporelle) associée à une dysfonction systolique ventriculaire gauche c′est-à-dire une fraction d’éjection du ventricule gauche inférieure (FEVG) à 0,45 et/ou une fraction de raccourcissement < 30%.

Nous avons recensé 48 cas suspects de la CMPP. Nous avons récusé 7patientes dont 3 présentaient des valvulopathies mitrales organiques, 2 avaient un échodoppler normal et 2 autres patientes étaient séropositives au VIH. Ainsi nous avons un échantillon de 41 patientes qui répondaient à tous nos critères

## Résultats

### Aspects épidémiologiques

Nous avons enregistré 41 cas de cardiomyopathies du péripartum en 27 mois, soit une moyenne de18 cas par an. La moyenne d’âge était de 31,47 ans (extrêmes de 21 et 44 ans). 60,97% patientes avaient 30 ans et plus. La CMPP représentait 1/362 naissances, 12,65% des IC totale et 22,65% des IC chez les femmes. Les CMPP représentaient 15,12% des cardiomyopathies dilatées et 26,11% cardiomyopathies dilatées chez les femmes. Les femmes sans emploi représentaient 65,85% de l’échantillon. Les femmes étaient de conditions socio-économiques défavorables dans 93,75%. Les multipares étaient les plus représentées avec 82,93%. La parité moyenne était de 3,07 (extrêmes de 1 à 6).

### Aspects cliniques

**Figure 1:**
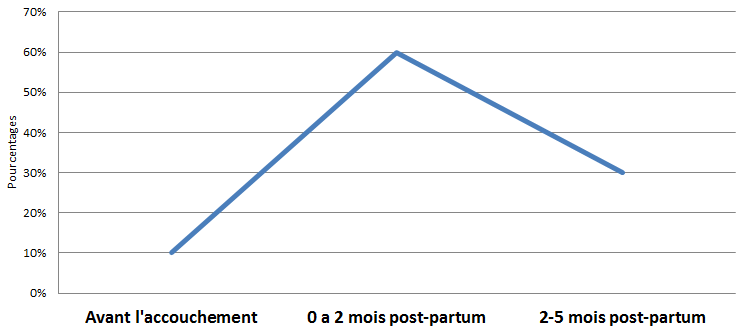
Début des symptômes par rapport à l'accouchement

Dans 90,24% les symptômes étaient apparus dans le post-partum (Figure 1). On notait un retard de consultation chez toutes les patientes avec un délai moyen de 53 jours (extrême: 06 à 120 jours). Les symptômes ont été dominés par la dyspnée d'effort (Tableau 1). La décompensation s’était faite sur le mode d'IC gauche isolée dans 34,15% des cas et sur le mode d'IC globale dans 65,85% des cas. Le Tableau 2 résume les données de l'examen physique.

**Tableau 1:** Les signes fonctionnels

	Nombre	Pourcentage%
**Dyspnée III**	29	70,13
**Dyspnée IV**	12	29,27
**Toux**	32	78,04
**OMI**	24	58,54
**Palpitations**	21	51,22
**Précordialgies**	15	36,58

**Tableau 2:** Les principales anomalies de l'examen physique.

	Nombre	Pourcentage%
**Tachycardie**	38	92,68
**Bruit de galop**	33	80,49
**Turgescence jugulaire**	25	60,96
**Souffle systolique**	23	56,09
**Hépatomégalie**	19	46,34
**Reflux hépato-jugulaire**	15	36,58

### Les signes radiographiques et électrocardiographiques

Une cardiomégalie a été retrouvée chez 39 patientes (95,12%) avec une moyenne de l'index cardiothoracique à 0,62 (extrême 0,52 à 0,8).

Une tachycardie sinusale de même qu'une hypertrophie ventriculaire gauche était notée chez 40 patientes (97,56%). Trente deux patientes (78,04%) avaient une hypertrophie auriculaire gauche et 58,54% des patients avaient une hypertrophie bi-ventriculaire. Il n'y avait pas de troubles de conduction auriculo-ventriculaire. Dans 29 cas (70,73%) on notait des troubles de repolarisation non spécifiques associés aux hypertrophies ventriculaires. L’électrocardiogramme était normal dans 1 cas

### Les données échocardiographiques

Le VG était dilaté chez toutes les patientes avec un diamètre télédiastolique allant de 34,2 à 46mm/m^2^ de surface corporelle (57,8 à 80mm). L'oreillette gauche était dilatée chez 37 patientes (90,24%), le ventricule droit était dilaté chez 26 patientes (63,41%). La dysfonction systolique du VG était constante et sévère. La fraction d’éjection du ventricule gauche (FE) était en moyenne de 0,34 (extrêmes 0,10 et 0,44). L'onde myocardique systolique (Sa) était abaissée chez toutes les patientes avec une moyenne de 0,47 (extrême 0,21 et 0,62). Toutes les patientes présentaient une hypokinésie pariétale globale. L’échodoppler cardiaque avait objectivé un thrombus intraventriculaire gauche chez 4 patientes (9,76%) et intraventriculaire droit chez 2 patientes (4,78%). Une insuffisance mitrale fonctionnelle était retrouvée dans 90,24%. Les pressions de remplissage du VG étaient augmentées chez 32 patientes (78,04%). L'hypertension artérielle pulmonaire était importante dans 56,09% des cas avec une moyenne de 57,37mmHg (extrêmes: 39 et 86mmHg).

### Evolution en hospitalisation

La durée moyenne d'hospitalisation de nos patientes était de 16 jours (extrêmes 7 à 33 jours). Trente deux patientes (78,04%) avaient retrouvé une guérison clinique avec disparition des signes, 3patientes (7,32%) avaient développé une insuffisance cardiaque irréductible. Nous avons noté 2 décès (4,88%).

## Discussion

L'incidence de la CMPP était de 1/362 dans notre étude. En Afrique l'incidence de la CMPP est variable [4, 5]. En occident la maladie parait moins fréquente avec une incidence 1/3000 à 1/15000 [6]. Ces résultats confirment que la CMPP est une pathologie qui sévit plus chez la femme d'origine noire [7, 8]. Les autres facteurs associés sont l’âge maternel avancé [9]. Dans cette étude, la parité moyenne était de 3,07 (extrêmes:1 et 6) avec 82,93% de multipares. Ceci témoigne de la fréquence de cette affection chez la femme multipare [9]. De plus 93,75% des patientes étaient de conditions socio-économiques faibles. Nous partageons avec d'auteurs que le bas niveau socio-économique est aussi un facteur de risque à la CMPP [10].

Au total, l’âge maternel supérieur à 30ans, la multiparité, les conditions socio-économiques défavorables constituent les facteurs de risque de la CMPP retrouvés dans cette étude. Les autres facteurs comme la notion d'HTA chronique et d'usage prolongée de tocolytiques, les grossesses gémellaires [9] ne peuvent pas être formellement retenues dans cette étude.

Nous avons observé un grand retard de consultation chez nos patientes. La dyspnée d'effort était le maître symptôme avec un stade avancé (classification NYHA). Les mêmes constats étaient rapportés dans la littérature africaine [4, 5, 10]. Ce sont des patientes en réalité chez qui les symptômes débutent plus tôt mais l'ignorance et la pauvreté seraient les causes de retards de consultation et la plupart des patientes sont retrouvées dans un tableau d'IC globale avec un état d'anasarque. Les femmes considéraient les ‘dèmes des membres comme fait normal lié à une grossesse et c'est devant l'intensité croissante de la dyspnée que la majorité avait consulté. Les autres symptômes retrouvés étaient les précordialgies et la toux. Les précordialgies allaient du simple picotement précordial à la douleur d'allure angineuse avec sensation d'oppression thoracique. Leur fréquence varie en fonction des auteurs [5, 8, 10]. Ces douleurs thoraciques associées à la toux posent un réel problème diagnostique car pouvant faire suspecter une embolie pulmonaire. Dans tous les cas les patientes sont suffisamment mises sous anticoagulant à dose curative. La tachycardie avec un bruit de galop, le souffle systolique d'insuffisance mitrale et les râles crépitants étaient les données auscultatoires les plus fréquentes retrouvées chez nos patientes. Plusieurs auteurs ont rapporté ces mêmes données de l'examen physique mais à des taux très variables [4, 5, 10]. Ces disparités statistiques s'expliquent par le caractère subjectif des examens cliniques d'où la nécessité de faire des examens paracliniques. La cardiomégalie était notée dans 95,12% des cas dans cette étude. La cardiomégalie est constante dans l'insuffisance cardiaque mais reste non spécifique [11]. Il s'agit d'un élément essentiel dans nos régions où l’échodoppler cardiaque est rare et inaccessible à la population.

A l'ECG, des troubles du rythme graves sont rapportés dans la littérature. Ferrière [12] sur 11 observations a noté 1cas de tachycardie ventriculaire. Il s'agit d'une tachycardie ventriculaire dépistée par enregistrement holter ECG. La tachycardie sinusale, l'HVG et les troubles de la repolarisation non spécifiques sont les anomalies électriques fréquemment retrouvées [5, 10].

Devant une accouchée récente qui se plaint d'une dyspnée, la découverte d'une cardiomégalie associée à une tachycardie sinusale et une HVG, doit faire retenir la CMPP jusqu’à preuve du contraire. L’échodoppler cardiaque ne viendra que pour confirmer le diagnostic et apprécier le retentissement et les complications.

Les signes échocardiographiques sont un des critères de définition de la CMPP et l'hypokinésie pariétale globale est le trouble constant retrouvé [2, 12]. La dilatation cavitaire de même que la dysfonction systolique de VG étaient sévères chez nos patients. Il s'agit des conséquences du retard de consultation et de diagnostic. La CMPP est une pathologie hautement emboligène [7, 13]. Les raisons évoquées sont multiples: une hypercoagulabilité sanguine au cours de la grossesse [14], une cardiomyopathie dilatée qui apparait chez une accouchée récente, une mobilité maternelle réduite pendant les derniers mois de la grossesse, une compression de la VCI par le mobile f'tal. Toutes ces raisons justifient le traitement anticoagulant curatif chez nos patientes.

L’évolution de la CMPP est imprévisible [15]. L'intervalle inter-génésique dépend du délai de retour de la fonction systolique à la normale. Lorsque l'IC persiste au-delà du sixième mois après accouchement, la mortalité est de 28% en un an et de 85% en 5 ans [2, 3, 16]. Les formes rebelles au traitement médical représenteraient 10% des cas [15]. Lorsque la CMPP est guérie, le risque de récidive lors d'une grossesse ultérieure ne peut être exclu. Les conseils à donner aux patientes sont donc adaptés à chaque cas. Certains éléments sont considérés comme de mauvais pronostic. Il s'agit de l'origine africaine, l’âge supérieur à 30 ans, un délai d'apparition des symptômes supérieur à 3mois, la persistance des signes cliniques 6 mois après le début de la maladie, un ICT supérieur à 0,6 et les caractéristiques du ventricule gauche: une dilatation peu importante (DTDVG< 55-60mm), une fraction d’éjection < 30%, une fraction de raccourcissement inférieur à 20% au moment du diagnostic [2, 14, 17]. Si nous considérons ces facteurs, nous dirons que toutes nos patientes présentaient un mauvais pronostic.

Le pronostic de la maladie est imprévisible. De nombreuses patientes décèdent malgré le traitement alors que d'autres évoluent tout à fait favorablement et on assiste après 6 a 12 mois de traitement à la guérison complète [2, 14, 17, 18]. Entre la guérison et le décès, l’évolution est celle d'une insuffisance cardiaque chronique d'une CMD [19]. Le pronostic obstétrical est médiocre. Une défaillance cardiaque survient dans 50 à 80% des cas lors des grossesses ultérieures, avec une mortalité qui peut atteindre 60% [18, 20]. Etant donné une mortalité très élevée lors des grossesses ultérieures nous avons en accord avec nos patientes multipares opté pour une contre indication de grossesse définitive. Les primipares désireuses d'avoir une autre grossesse sont suivies et les décisions seront données cas par cas.

## Conclusion

La cardiomyopathie du péripartum est une complication cardiaque grave de la grossesse. Elle est fréquente au Togo comme dans les autres pays d'Afrique noire. Elle survient préférentiellement dans le post partum. Les facteurs de risque étaient: l’âge maternel supérieur à 30ans, la multiparité et les conditions socio-économiques défavorables. On observait un retard de diagnostic important. Le tableau clinique était celui d'une insuffisance cardiaque globale avec dilatation importante des cavités cardiaques et des altérations sévères des performances myocardiques.
